# 3-Tesla T2 Mapping Magnetic Resonance Imaging for Evaluation of SLAP Lesions in Patients with Shoulder Pain: An Arthroscopy-Controlled Study

**DOI:** 10.3390/jcm12093109

**Published:** 2023-04-25

**Authors:** Patrick Stein, Felix Wuennemann, Thomas Schneider, Felix Zeifang, Iris Burkholder, Marc-André Weber, Hans-Ulrich Kauczor, Christoph Rehnitz

**Affiliations:** 1 Diagnostic and Interventional Radiology, University Hospital Heidelberg, Im Neuenheimer Feld 420, 69120 Heidelberg, Germany; 2 Institute of Diagnostic and Interventional Radiology & Neuroradiology, Helios Dr. Horst Schmidt Clinics Wiesbaden, Ludwig-Erhard-Straße 100, 65199 Wiesbaden, Germany; 3 Center for Orthopedics, Trauma Surgery and Spinal Cord Injury, University Hospital Heidelberg, Schlierbacher Landstraße 200A, 69118 Heidelberg, Germany; 4 Ethianum Clinic Heidelberg, Voßstraße 6, 69115 Heidelberg, Germany; 5 Department of Nursing and Health, University of Applied Sciences of the Saarland, 66117 Saarbruecken, Germany; 6 Institute of Diagnostic and Interventional Radiology, Pediatric Radiology and Neuroradiology, University Medical Center Rostock, Ernst-Heydemann-Straße 6, 18057 Rostock, Germany

**Keywords:** T2 mapping, glenoid labrum, superior labral anterior posterior (SLAP) lesions, arthroscopy

## Abstract

This study investigated the ability of T2 mapping to assess the glenoid labrum and to differentiate between healthy labral substances and superior labral anterior posterior (SLAP) lesions using arthroscopy as the gold standard. Eighteen patients (mean age: 52.4 ± 14.72 years, 12 men) with shoulder pain were examined using 3-Tesla T2 mapping. All the patients underwent shoulder arthroscopy. Using morphological sequences for correlation, regions of interest covering the entire labral substance were placed in the corresponding T2 maps. The diagnostic cutoff values, sensitivities, and specificities, as well as the inter-reader correlation coefficients (ICCs) determined by two independent radiologists, were calculated. The mean T2 value was 20.8 ± 2.4 ms for the healthy labral substances and 37.7 ± 10.63 ms in the patients with SLAP lesions. The maximum T2 value in normal labrum (21.2 ms) was lower than the minimum T2 value in the patients with SLAP lesions (27.8 ms), leading to sensitivities, specificities, and positive and negative predictive values of 100% (95% CI 54.1–100.0) for all the cutoff values between 21.2 and 27.8 ms. The ICCs ranged from 0.91 to 0.99. In summary, the data suggest that evaluation and quantification of the labral (ultra)structural integrity using T2 mapping may allow discrimination between arthroscopically confirmed SLAP lesions and a healthy glenoid labrum. T2 mapping may therefore be helpful in diagnosing patients with suspected labral damage.

## 1. Introduction

Shoulder pain is a significant medical and socioeconomic problem that can lead to the inability to work and perform household or leisure activities. In a systematic review, Luime et al. reported a one-year prevalence of shoulder pain of up to 46.7% and a lifetime prevalence of up to 66.7% [1]. In addition, an age-dependent distribution has been reported, with older people being more likely to experience shoulder pain [1]. One repeatedly encountered source of shoulder pain affecting all age groups is superior labral anterior posterior (SLAP) lesions. The superior labrum plays an important role in the stability of the shoulder joint. Accordingly, SLAP lesions have been documented to cause both anterior and posterior instabilities in overhead athletes [2,3,4]. Microinstability, in turn, can lead to various shoulder injuries, including cartilage defects, and may progress to advanced osteoarthritis (OA) [5,6]. Despite the increasing understanding of its pathomechanisms and epidemiology, the diagnosis of superior labral lesions remains clinically and radiologically challenging.

Over the last few decades, the incidence of SLAP lesions has increased [7,8]. A study based on private insurance data from 2003 to 2013 reported a fivefold increase in the incidence of SLAP lesions [9]. In the younger population, traumatic shoulder injuries and participation in sports with overhead throwing movements are major causes of superior labral lesions, whereas chronic degeneration seems to play a major role in the development of SLAP lesions in older adults [10,11]. In 53 asymptomatic middle-aged subjects (45–60 years), non-contrast magnetic resonance imaging (MRI) revealed a prevalence of up to 72% for superior labral tears [12]. A similar study found no labral tears but observed an “abnormal labral signal” in 50% of a symptomatic subgroup in a comparatively younger cohort (25–55 years) [13]. However, in neither of these studies was the diagnosis confirmed via arthroscopy. Furthermore, histological post-mortem studies showed marked degenerative changes in the labral substance as well as the more frequent occurrence of labral tears with increasing age [14,15]. Thus, the continuous transition of age-related degenerative changes into manifest labral tears appears to be a relevant pathomechanism.

Along with the increase in incidence, there has been an increase in the number of surgically treated labral lesions [7,8,16]. While some studies have shown satisfactory postoperative outcomes [17,18], several recent studies have reported complications and the incomplete regression of symptoms, particularly in middle-aged and older patients, as well as in professional overhead athletes [8,19,20,21]. Therefore, timely diagnosis of early and potentially reversible damage to the collagen matrix is desirable to delay or prevent the clinical progression of manifest labral tears.

MRI, which is sometimes supplemented by MR arthrography, is the radiological gold standard for the morphological evaluation of the glenoid labrum [22,23]. The diagnostic performance of non-contrast MRI in superior labral tears has been studied several times, with variable results in terms of accuracy and sensitivity [24,25]. While some studies have reported high diagnostic accuracy for MR arthrography, others have demonstrated non-specific as well as non-sensitive results for MRI and MR arthrography in the diagnosis of SLAP lesions [26,27,28]. Early degenerative changes usually cannot be detected with conventional morphological MRI sequences; however, functional sequences such as delayed gadolinium-enhanced MRI of cartilage (dGEMRIC), T2* mapping, and T2 mapping allow the assessment of the biochemical composition of joint structures [29,30,31]. This enables the detection of damage to the ultrastructural integrity of cartilage or collagen networks. T2 mapping can detect and quantify early degenerative changes in the cartilage of numerous joints, including the wrist, ankle, metacarpophalangeal joints, and knee [29,32,33,34]. As high T2 values reflect disruption of the 3D collagen network and an increase in water content, T2 mapping may also be appropriate for fibrocartilaginous or collagenous structures such as the glenoid labrum and menisci, respectively. In knee OA, a correlation between high T2 values and histologically confirmed signs of meniscal degeneration was demonstrated [35]. However, the study included patients with severe OA who were scheduled for knee arthroplasty, indicating a high pre-test probability of concomitant meniscal degeneration.

To our knowledge, no studies to date have evaluated the diagnostic performance of T2 mapping for the assessment of the glenoid labrum. In addition, T2 mapping of the shoulder joint in general has hardly ever been studied, and only a few existing studies have validated this technique using arthroscopy.

Thus, the purpose of this study was to evaluate the ability of T2 mapping to assess the biochemical integrity of the superior labrum and to differentiate between individuals with and without SLAP lesions in patient cohorts with shoulder pain but not high-grade OA, using shoulder arthroscopy as the gold standard. As a secondary study aim, the interrater reliability was assessed by calculating the intraclass correlation coefficient (ICC).

## 2. Materials and Methods

### 2.1. Participants

This study included patients with shoulder pain who were to undergo arthroscopy and were referred to our department for preoperative MRI over a period of 3 months. The exclusion criteria were as follows: endoprosthetic replacement of the glenohumeral joint or osteosynthetic material involving the proximal humerus; prior tendinous or ligamentous refixation procedures; advanced OA (Kellgren–Lawrence score > 1); and age less than 18 years. Nineteen consecutive patients who met the inclusion criteria were enrolled in the study initially. As one patient, in whom only a routine MRI protocol without T2 mapping sequences was inadvertently applied, had to be excluded, 18 patients remained for inclusion.

### 2.2. Study Design and Reporting

After inclusion into the study, the index test (3-Tesla MRI with T2 mapping) was performed in each subject. After a maximum interval of 6 days (mean interval: 4 days) the reference test (shoulder arthroscopy) was carried out. Subsequently, the subgroups were divided into “healthy individuals” and “patients with proven SLAP lesion” depending on the arthroscopy findings. Therefore, this study can be defined as a one-stage, case–control study according to item 5 of the STARD guidelines published by the EQUATOR Network in 2015 [36].

### 2.3. MRI Protocol and T2 Mapping

The 3-Tesla MRI system used in this study was a Magnetom Verio (Siemens Healthineers, Erlangen, Germany) with a 70 cm gantry width and an 18-channel total imaging matrix. To ensure that the shoulder was as close as possible to the magnetic isocenter, all the patients were placed in a supine position with the head first and with the shoulder joint stabilized in external rotation. Proton density-weighted, fat-saturated MRI sequences as well as T1- and T2-weighted sequences without fat saturation were used for morphological assessment of the (fibro)cartilaginous joint structures. Oblique coronal and oblique sagittal sequences were oriented perpendicular and parallel to the glenoidal fossa, respectively. A multi-echo spin-echo sequence for inline T2 mapping provided by the manufacturer (syngo MapIT, Siemens Healthineers, Erlangen, Germany) was used as the study sequence. In this sequence, a pixel-wise, monoexponential, non-negative least squares fit analysis was used to derive the T2 relaxation times from the T2 parameters, which were then used for further analysis. A color-coded map was automatically generated from the quantitative T2 relaxation times. A detailed overview of the in-house shoulder protocol and the T2 mapping study sequence is given in Table 1.

### 2.4. Image Analysis and Definition of SLAP Lesions

The morphological sequences were evaluated using a picture archiving and communication system (Centricity PACS, v. 4.0; GE Healthcare IT Solutions, Barrington, IL, USA). Two independent musculoskeletal radiologists with 19 (CR) and 6 (FW) years of experience in musculoskeletal MRI evaluated the morphological as well as the color-coded study sequences. Both readers had participated in previous training sessions on the morphological analysis of the glenoid labrum, which included cases beyond the scope of this study. In addition, both readers had experience in the evaluation of quantitative biochemical imaging techniques, particularly for cartilaginous structures. Both radiologists were blinded to the results of the arthroscopy, the clinical data of the patients, and the patients’ names. There was no blinding of the study participants, who had been informed about the additional study sequences and who knew that they were scheduled for shoulder arthroscopy. In accordance with the retrospective, one-stage, case–control study design, randomization was not performed.

Both readers set parameters for slice selection, magnification, and windowing. During the reading sessions, the ambient light was reduced to a minimum.

The glenoid labrum was classified as either normal or damaged using morphological proton density-weighted, fat-saturated sequences. Following Snyder et al., irregular labral margins with increased signal intensity, stripping of the superior labrum from the glenoid, and a bucket-handle tear of the superior labrum, with or without extension into the origin of the long biceps tendon, were defined as SLAP lesions [37]. If none of these diagnostic criteria was met, the labrum was considered to be normal.

### 2.5. Placement of Regions of Interest

**Figure 1:**
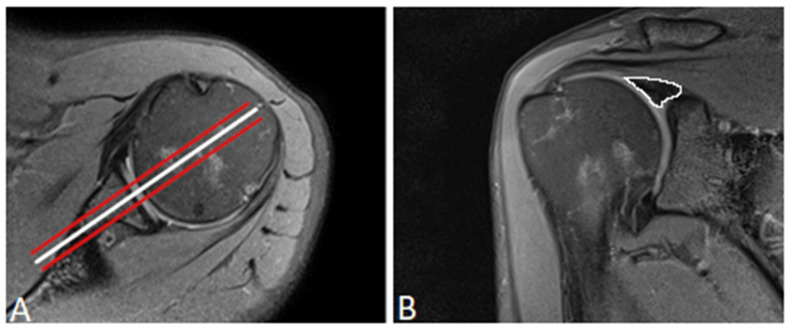
(**A**) Axial proton density-weighted, fat-saturated magnetic resonance image demonstrating the three oblique coronally oriented sections used for ROI placement: one midsection through the glenoid fossa (white) as well as one adjacent slice, anteriorly and posteriorly (red). (**B**) Coronal proton density-weighted, fat-saturated magnetic resonance image depicting the manually drawn ROI (white) outlining of the entirety of the glenoid labrum on a midcoronal section. The ROI was copied into the color-coded T2 mapping sequence.

First, we used the proton density-weighted fat-saturated sequences to delineate the superior labrum and determine the optimal position for region of interest (ROI) placement. The first ROI was placed in the midsection of the glenoid fossa, where the labrum had the largest dimension and appeared triangular. To reduce the effect of artifacts or imperfect ROI placement, two additional ROIs were placed, one in the anterior slice and one in the posterior adjacent slice, leaving three sections for ROI placement (Figure 1). To provide the most accurate measurement results, the ROIs were first placed in the first echo images acquired from the multi-echo spin-echo T2-weighted sequences, which further supported the morphological delineation of the labral substance. A careful visual comparison with fat-suppressed, proton density-weighted sequences was performed to ensure that the entire labrum was captured. In the next step, the ROIs were copied into the color-coded parametric T2 map. Where necessary, the copied ROIs were adjusted to ensure that the final ROI did not include any artifacts, glenoid cartilage or cortex, joint capsule, supraspinatus muscle, or synovial fluid. The average T2 relaxation times of the three adjacent ROIs were used for further evaluation.

The presence or absence of SLAP lesions in the morphological proton density fat-saturated (PDfs) sequences did not influence the placement of the ROIs, as the whole dimension of the labrum was always analyzed and compared with the arthroscopy. However, we also assessed the presence or absence of SLAP lesions in the morphological PDfs sequences and compared the findings with those of the arthroscopy.

### 2.6. Arthroscopy

Shoulder arthroscopy was performed by one of the two experienced orthopedic surgeons. According to the in-house standard, the arthroscopies were performed under general intravenous anesthesia, with all the patients placed in the beach-chair position. A posterior approach was selected for diagnostic inspection of the glenoid joint. The glenoid labrum, glenoid cartilage, and intra-articular portion of the long biceps tendon were assessed using a standardized questionnaire. The labral lesions were classified according to the method described by Snyder et al. [37]. A ventral approach was established if surgical intervention was required.

### 2.7. Statistical Analysis

The demographic data were analyzed descriptively. The continuous variables were summarized as mean, standard deviation, median, minimum, and maximum. For the qualitative variables, the frequency and percentage were analyzed. The patients’ ages in the groups with and without lesions were compared using the exact Wilcoxon two-sample test.

The T2 imaging analysis was conducted in a descriptive manner using summary statistics and was interpreted exploratively. The Shapiro–Wilk test was used to determine whether the T2 mapping parameters were normally distributed. The groups with and without labral lesions were compared using two-sample t-tests, with the level of significance set at 5%.

The diagnostic performance of the T2 mapping for SLAP lesions was described using estimates and exact 95% confidence intervals for sensitivity (proportion of true positive patients out of all patients with SLAP lesions); specificity (proportion of true negative patients out of all patients without SLAP lesions); and positive (proportion of true positive patients out of all positive patients) and negative predictive values (proportion of true negative patients out of all negative patients).

The intraclass correlation coefficient (ICC) was used to analyze inter-reader agreement. The two readers were treated as a random sample of observers from a larger population of potential observers. According to Shrout and Fleiss (1979), for interrater reliability a two-way random effects model with subject and rater as random effects was applied for the estimation of the ICC and 95% confidence intervals [38]. All statistical analyses were performed using SAS for Windows (version 9.4; SAS Institute Inc., Cary, NC, USA) and R version 3.5.1 (www.cran.r-project.org (accessed on 10 May 2022)).

## 3. Results

### 3.1. Demographic Data

Of the 18 patients included, 12 (66.7%) were male and 6 (33.3%) were female. The mean patient age was 52.4 ± 14.72 (range, 22.0–67.0) years. Patients without SLAP lesions were only slightly younger than those with SLAP lesions (46.2 ± 18.82 [range, 22–64] years vs. 55.5 ± 11.93 [range, 29–67] years). Of the 12 patients with arthroscopically confirmed SLAP lesions, 9 (75%) were male and 3 (25%) were female. Table 2 summarizes the demographic characteristics of the patients according to the presence or absence of SLAP lesions.

### 3.2. Arthroscopic Evaluation of the Glenoid Labrum

Shoulder arthroscopy was performed in all patients, with a mean interval of 4 days between MRI examination and surgery (range, 0–6 days). A diagnosis of SLAP lesions was made in 12 patients (66.7%). Following Snyder et al., nine SLAP lesions were classified as type 1 (75.0%), one as type 2 (8.3%), and two as type 3 (16.7%). No SLAP lesions were diagnosed as type 4 (Table 3).

Several patients with SLAP lesions were diagnosed with concomitant shoulder pathologies at arthroscopy. Focal cartilage damage was seen in 5 of the 12 patients diagnosed with SLAP lesions (41.7%), and damage to the long biceps tendon was seen in 6 of the 12 patients (50.0%). In addition, 2 out of the 12 patients (16.7%) were diagnosed with all three entities: SLAP lesion, focal cartilage damage, and lesion of the long biceps tendon.

### 3.3. T2 Mapping of the Glenoid Labrum

**Figure 2:**
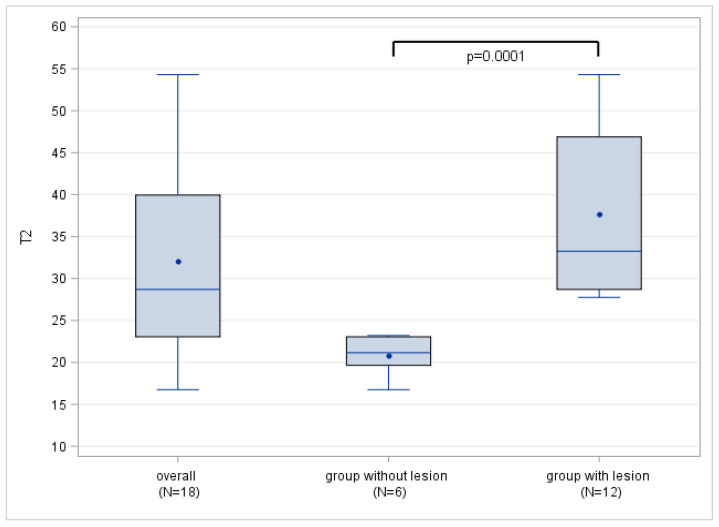
Boxplots of overall T2 mapping values (ms) in those with a healthy labrum and in patients with SLAP lesion. Note the complete separation of T2 values between individuals with SLAP lesion and those with a healthy labrum. The blue horizontal lines inside the box plots represent the median, the blue dots the mean T2 value.

Twelve SLAP lesions were detected by MRI, which corresponded with the number of lesions detected arthroscopically. No additional lesions were observed. Table 4 shows the mean T2 mapping parameters for patients with and without SLAP lesions. The mean T2 values were 37.7 ± 10.63 ms for the patients with SLAP lesions and 20.8 ± 2.40 ms for the population without labral damage, showing a significant difference between the two groups (*p* < 0.0001). There was complete separation of the T2 mapping values between the normal and damaged glenoid labrum, as the maximum T2 value for the group without lesions (21.2 ms) was lower than the minimum T2 value for the patients with SLAP lesions (27.8 ms; Figure 2 and Table 4). Therefore, all the cutoff values between these two values yielded the same values for the sensitivity and positive predictive value of 100% with 95% CI (73.5–100.0%) and for the specificity and negative predictive value of 100% with 95% CI (54.1–100.0%). ICC analysis revealed near-perfect inter-reader agreement of 0.97 (95% CI 0.91–0.99).

**Figure 3:**
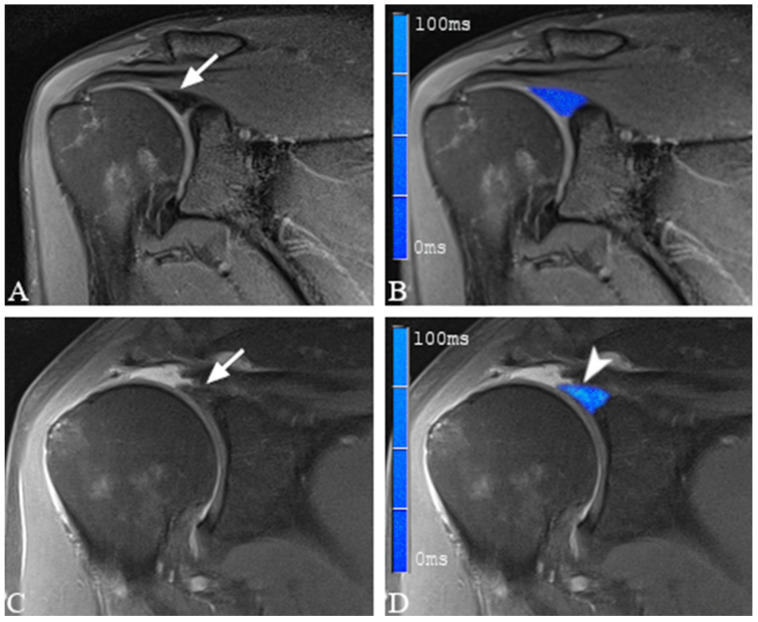
(**A**,**C**) show coronal proton density-weighted, fat-saturated magnetic resonance images of a 34-year-old woman with morphologically normal-appearing and arthroscopically proven healthy labrum (arrow in (**A**)), as well as fraying of the superior labrum in a 57-year-old man with arthroscopically proven SLAP lesion Type 1 (arrow in (**C**)). (**B**,**D**) show merged images of the proton density-weighted images and the corresponding color-coded T2 maps. Note the elevated average T2 mapping value of 53.9 ms in the damaged labral substance (arrowhead in (**D**)). The average T2 mapping value for the healthy labral substance was 21.3 ms (**B**).

**Figure 4:**
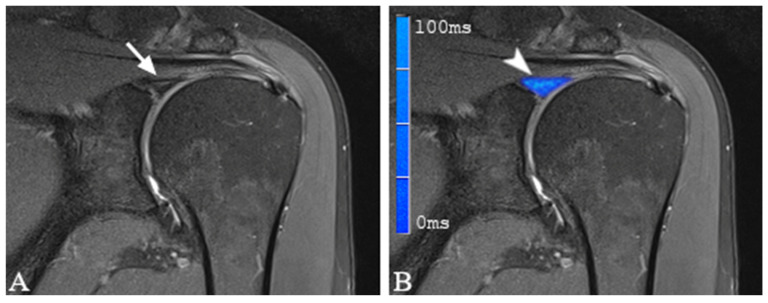
(**A**) shows a coronal proton density-weighted, fat-saturated magnetic resonance image of a 62-year-old man with linear hyperintensity centrally within the glenoid labrum indicating labral damage (arrow). (**B**) shows a merged image of (**A**) and the corresponding color-coded T2 map. Note the elevated average T2 mapping value of 40.5 ms (arrowhead). During arthroscopy a SLAP lesion Type 2 was present.

Color-coded T2 maps depicted a visual difference between healthy and damaged labral substance (Figure 3 and Figure 4).

## 4. Discussion

Despite the increasing incidence over the past two decades and the improved understanding of the predisposing factors and the pathomechanism, the diagnosis of SLAP lesions remains difficult both clinically and by MRI. Early diagnosis of the pathologies of the glenoid labrum and the associated changes in biochemical composition using MRI has not yet been established, but it is desirable, for instance, to initiate therapy or modify training plans at an early stage. T2 mapping is an MRI technique that has proven to be effective in assessing the integrity of the collagen network and water content of articular cartilage in various joints [29,32,33,34]. However, this technique has not yet been systematically used to assess the glenoid labrum, a structure which is composed predominantly of fibrocartilaginous tissue organized in a 3D collagen network, and correlations with arthroscopy as the gold standard are lacking.

Thus, this study evaluated the ability of T2 mapping to detect and quantify labral damage containing SLAP lesions in patients with shoulder pain and to differentiate damaged labra from normal labra using shoulder arthroscopy as the gold standard. The patients with SLAP lesions had a significantly higher mean T2 value than those without (37.7 ± 10.63 ms vs. 20.8 ± 2.40 ms; *p* < 0.0001). The increase in T2 values may have been due to damage to the ultrastructural architecture of the collagen network and the associated repair mechanisms. While the exact histological composition of the glenoid labrum is still controversial, it has been shown to consist primarily of fibrocartilaginous tissue that transitions into hyaline articular cartilage towards the glenoid [39,40,41]. Several studies have identified loss of the collagenous matrix, with the corresponding areas of increased cellularity, vascularity, and water content, as histological correlates for degeneration in cartilaginous and fibrous tissue [42,43,44,45]. These biochemical changes are consistent not only with the increased T2 values of the labrum observed in our study but also with several other studies reporting increased T2 values in damaged articular cartilage or tendons [46,47,48]. While hyperintense patterns in T2-weighted images of the damaged glenoid labrum have been reported before, this is to our knowledge the first study quantifying the actual T2 relaxations times using T2 mapping [49,50]. Thus, by providing objectifiable measurements, this technique may be useful for establishing diagnostic cutoffs, or it may serve as a longitudinal biomarker for healing processes. Further studies with larger sample sizes are needed to clarify the extent to which a change in T2 relaxation times correlates with actual lesion severity.

In our study population, the highest overall T2 value in the healthy patients (21.2 ms) was lower than the lowest overall T2 value in the patients with SLAP lesions (27.8 ms). This amounts to a complete separation of the data between the two groups. Thus, all the diagnostic cutoff T2 values between 21.2 and 27.8 ms would result in the sensitivity, specificity, positive predictive value, and negative predictive value of 100% (95% CI 54.1–100.0). As this is, to our knowledge, the first study to systematically evaluate T2 relaxation times using T2 mapping of the glenoid labrum, no appropriate data are available for direct comparison. Nevertheless, the mean T2 mapping values found in our study for healthy individuals (20.8 ± 2.4 ms), as well as for those for damaged labral substance (37.7 ± 10.63 ms), are similar to the reported T2 mapping values for the articular cartilage of the glenoid. One study described average T2 mapping values of 23.0 ± 3.0 ms for healthy glenoid cartilage and 44.8 ± 3.7 ms in patients with focal cartilage defects, thus demonstrating comparable quantitative differentiation [51]. The minor variations of the T2 mapping values may reflect the differences in histological composition, with mainly fibrocartilaginous tissue found in the labrum compared to the mainly hyaline articular cartilage.

Several studies have reported significant differences in T2 mapping values between normal and damaged cartilage [47,48,52,53]. Because the glenoid labrum is composed primarily of (fibro)cartilaginous tissue, its T2 mapping value is subject to a variety of factors known to influence the signal intensity of the articular cartilage. In particular, differences in the mechanical load, with the associated variations in water content and matrix composition, as well as the artificial signal changes such as the “magic angle” effect, have been described [31,45,54]. The latter is known to increase the T2 relaxation time in collagen fibers oriented at 55° to the magnetic field, especially when using short echo times (TE) [55,56,57]. In a retrospective arthroscopy-controlled study, Sasaki et al. reported increased signal intensity of the posterosuperior labrum, not only in patients with confirmed labral damage, but also in healthy individuals [58]. When a group of six asymptomatic participants was studied, the same group showed signal changes in the posterosuperior labrum, depending on the TE and positioning of the volunteers [58]. Thus, because the magic angle artifact would also cause signal alterations in healthy labral substances, complete separation of the data cannot be explained by artificial means alone. Our results could be due to the precise placement of the ROI, as well as the small study population with only six patients without SLAP lesions. Future studies with larger sample sizes may show an overlap of T2 mapping values, indicating a continuous progression of degenerative labral changes manifesting as SLAP lesions.

The T2 mapping values for the normal and damaged cartilage showed near-perfect interrater agreement, with respective ICCs of 0.97 (95% CI 0.91–0.99), which may be interpreted as a promising sign for the reliable application of this technique in clinical practice. These findings are consistent with the previously published studies of T2 mapping of the glenoid joint in healthy subjects, reporting good to excellent interrater agreement [59,60]. However, considering the small sample size and the associated small number of patients with SLAP lesions (*n* = 12), our results should be interpreted with caution and could be at least partially explained artificially.

While this study focused on differentiating labra containing SLAP lesions from the normal glenoid labrum, T2 mapping may also detect early degenerative changes that are not readily apparent in morphological sequences. In a post-mortem study, Pfahler et al. investigated histological changes in the labral substance in different age groups and reported a significant increase in the number and severity of micro- and macrolesions in the labrum with increasing age [14]. Additionally, Schwartzberg et al. reported a prevalence of up to 72% for superior labral tears in asymptomatic middle-aged individuals [12]. This finding is comparable with the 66.7% prevalence of SLAP lesions observed in our symptomatic study population. The continuous transformation of early degenerative changes into manifest labral lesions with increasing age may be accentuated by individual (micro)traumas, which could be a possible pathomechanism for the development of SLAP lesions. The detection of early degenerative changes could allow the timely initiation of protective conservative measures, such as physical therapy, to delay or prevent further disease progression. Future studies should investigate the extent to which differing T2 mapping values in morphologically normal-appearing labrums can be interpreted as an indicator of early degenerative changes. T2 mapping could then be used to assess the effect of early conservative interventions, such as physiotherapy. Additionally, several studies have shown that T2 mapping is effective in monitoring biochemical changes after cartilage repair procedures [61,62]. Xie et al. reported a correlation between a decrease in T2 mapping values and an improvement in clinical outcomes in the first year after arthroscopic rotator cuff repair, further highlighting the potential clinical utility of this technique [46].

Our study has some limitations. The small sample size may have contributed to the complete separation of T2 mapping values between the normal and damaged labral substances, resulting in a statistically perfect discrimination. In particular, the wide confidence interval (CI) of 54.1 to 100%, which is at least partly due to the small sample size, has several statistical implications. First, the small sample size affects the calculation of the confidence interval (CI) by overweighting extreme values, thus resulting in a wider CI range. Second, the sample size was too small to allow a statistically valid distinction between the different types of SLAP lesions. By generalizing the spectrum of lesion severity as “pathologic” versus “physiologic”, the statistical calculation is susceptible to sampling biases such as spectrum bias, in which a study population has a different spectrum of lesion severity compared with the normal population. This is particularly important because the patient cohort was recruited at a tertiary referral center and was therefore highly selective, which may have affected the prevalence of SLAP lesions; this, in turn, may have had an effect on the sensitivity and specificity values. A patient population with a higher prevalence of SLAP lesions may include more patients with higher lesion grading, making the test perform better in this population. In view of these statistical limitations, as well as the above-mentioned variations in T2 values, our results may only be applicable to our patient cohort. Studies with larger sample sizes that distinguish between levels of lesion severity are needed to clarify this issue.

The large age discrepancy (22.0–67.0 years) must be considered a significant limitation, especially given the statistical implications of the small sample sizes discussed above. Although the age range was quite similar between the healthy subjects (22.0–64.0 years) and the patients with SLAP lesions (29.0–67.0 years), further studies with larger samples are required to allow better differentiation between age groups and to thereby address the process of age-related degeneration and the dehydration of cartilaginous structures beginning at 45–60 years of age.

Moreover, as this is the first study to quantify T2 relaxation times using T2 mapping in the glenoid labrum, there are no available data for a direct comparison of the labral T2 values.

Additionally, as mentioned above, the subgroup of patients with SLAP lesions was too small for statistically valid differentiation between SLAP types 1–4. The choice between surgical or conservative therapy and between the different surgical procedures depends largely on the attributed SLAP type [63,64]. Further studies with larger sample sizes may reveal significant differences in the T2 mapping values between SLAP types and might help to distinguish between conservatively treatable lesions and those requiring surgery.

We acknowledge that manual ROI placement in correlation with morphological MRI sequences is prone to human error. Although we reduced the impact of the potentially imprecise ROI placement by averaging three adjacent ROIs, high-resolution, three-dimensional T2 mapping sequences that can examine the entire labral substance are desirable for further studies as they will allow the voxel-wise evaluation of T2 mapping values. Additionally, using our method of ROI placement by averaging the T2 mapping values of midcoronal and anterior and posterior adjacent slices, lesions in a very anterior or very posterior position could have been missed. Therefore, three-dimensional T2 mapping sequences would allow for an even more precise assessment in future studies.

## 5. Conclusions

T2 mapping can evaluate and quantify the biochemical integrity of labral substances and differentiate between arthroscopically confirmed SLAP lesions and the healthy glenoid labrum. In addition to the mere morphological evaluation of the labrum, the quantification of T2 relaxation times using T2 mapping may provide objectifiable measures for diagnostic cutoff values and also function as a biomarker for monitoring healing processes under conservative therapy or postoperatively. By also providing information on the (ultra)structural integrity of the (fibro)cartilaginous networks, T2 mapping could prove help in therapeutic decision making, not only in labral pathologies but in several shoulder pathologies in general. The inclusion of T2 mapping sequences in the routine MRI protocol for suspected fibrocartilaginous or tendinous lesions may allow accurate diagnosis without the need for more invasive diagnostic procedures such as MR arthrography or even arthroscopy.

## Figures and Tables

**Table 1:** In-house shoulder MRI protocol and T2 mapping study sequences.

No.	Sequence	Orientation	Repetition Time (TR; ms)	Echo Time (TE; ms)	Acquisition Matrix	Flip Angle	Echo Train Length	No. of Slices	TA (Min)
1	PD fs TSE	axial	3660	24	384 × 346	176	7	27	04:32
2	PD fs TSE	oblique coronal	2490	24	384 × 307	160	7	19	03:37
3	PD fs TSE	oblique sagittal	3950	23	320 × 256	140	7	29	04:49
4	PD TSE	oblique coronal	1670	23	384 × 307	160	5	19	03:24
5	T1 SE	oblique coronal	787	10	384 × 346	90	1	19	04:51
6	T2 TSE	oblique sagittal	5640	88	384 × 307	150	15	29	02:33
7	T2 MapIt	oblique coronal	2140	13.8, 27.6, 41.4, 55.2, 69	320 × 320	180	1	16	06:50

TA = time of acquisition; PD = proton density; fs = fat-saturated, TSE = turbo spin echo.

**Table 2:** Demographic characteristics of patients with and without SLAP lesions.

		SLAP Lesion		
		No (N = 6)	Yes (N = 12)	*p*-Value Wilcoxon
Gender	male	3 (50.0%)	9 (75.0%)	
	female	3 (50.0%)	3 (25.0%)	
Age (years)	*n*	6	12	0.2216
	Mean	46.2	55.5	
	SD	18.82	11.93	
	Median	53.5	58.5	
	Min	22.0	29.0	
	Max	64.0	67.0	

SD = standard deviation; Min = minimum; Max = maximum.

**Table 3:** Distribution of SLAP types according to the classification by Snyder et al.

		Total (N = 18)
SLAP lesion	no	6 (33.3%)
	yes	12 (66.7%)
SLAP Classification	SLAP 1	9 (75.0%)
	SLAP 2	1 (8.3%)
	SLAP 3	2 (16.7%)
	SLAP 4	--

**Table 4:** T2 mapping values (ms) for normal and damaged glenoid labrum.

		Overall	Population without Lesion	Population with Lesion
T2	*n*	18	6	12
	Mean	32.1	20.8	37.7
	SD	11.90	2.40	10.63
	Median	28.7	21.2	33.3
	Min	16.8	16.8	27.8
	Max	54.3	21.2	54.3

SD = standard deviation; Min = minimum; Max = maximum.

## Data Availability

The datasets generated and analyzed in the present study are available from the corresponding author on reasonable request.
